# Identification of LILRB4 as a regulator of M2c macrophages and a potential immunotherapeutic target in ovarian cancer

**DOI:** 10.3389/fimmu.2026.1866073

**Published:** 2026-07-27

**Authors:** Huijuan Zhou, Qing Zhang, Bo Yin, Lingfei Han, Xiaohui Huang, Shuangdi Li

**Affiliations:** 1 Department of Gynecology, Shanghai Key Laboratory of Maternal Fetal Medicine, Shanghai Institute of Maternal-Fetal Medicine and Gynecologic Oncology, Shanghai First Maternity and Infant Hospital, School of Medicine, Tongji University, Shanghai, China; 2 Department of Gynecology, Wuhan Children’s Hospital (Wuhan Maternal and Child Health care Hospital), Tongji Medical College, Huazhong University of Science & Technology, Wuhan, Hubei, China

**Keywords:** biomarker, LILRB4, M2 macrophage, macrophage polarization, ovarian cancer

## Abstract

**Introduction:**

Ovarian cancer (OC) is the most lethal gynecological malignancy. A deeper insight into tumor microenvironment (TME) interactions is essential for developing novel therapeutic approaches. leukocyte immunoglobulin-like receptor B4 (LILRB4) is a receptor involved in multiple biological and pathological processes in hematological malignancies; however, its role in the progression of solid tumors remains largely unexplored. In particular, the function within the OC is still unclear.

**Methods:**

We systematically investigated the expression profile of LILRB4 in OC, along with its regulatory effects on OC cells, its role in immune cell modulation, and its potential as an immunotherapeutic target, using bioinformatics analyses, *in vitro* functional assays, and an *in vivo* orthotopic tumor model.

**Results:**

We found that LILRB4 is the most highly expressed member of the LILRB family in OC, with significantly higher expression in tumor tissues than in normal ovarian tissues, and its elevated expression was associated with poor patient survival. *In vitro* functional assays demonstrated that LILRB4 knockdown significantly inhibited OC cell proliferation, migration, and invasion, whereas overexpression of LILRB4 promoted these malignant phenotypes. Further analyses revealed that LILRB4 expression was positively correlated with the infiltration of tumor-associated macrophages in the TME and promoted macrophage polarization toward the immunosuppressive M2c phenotype. Mechanistically, *in vitro* experiments showed that LILRB4 promoted tumor cell secretion of CCL5, activated the NF-κB p65 signaling pathway, and enhanced M2c macrophage polarization, thereby promoting tumor progression and immune suppression. Finally, we demonstrated that downregulation of LILRB4 could enhance the sensitivity of OC to immunotherapy.

**Discussion:**

These findings identify LILRB4 as a key regulator of tumor progression and immune evasion in OC. High LILRB4 expression is associated with an immunosuppressive TME and poor response to immunotherapy, highlighting its potential as both a prognostic biomarker and a therapeutic target.

## 1 Introduction

As the most lethal gynecological malignancy, ovarian cancer (OC) faces major challenges in clinical prevention and treatment (1, 2). Its high mortality is primarily attributed to the absence of specific early symptoms and delayed diagnosis, with approximately 70% of patients being diagnosed at an advanced stage and thus missing optimal treatment opportunities, resulting in an overall 5-year survival rate of only around 30% (3, 4). Epidemiological data indicate that the incidence of OC in China has been continuously increasing in recent years, with a trend toward younger onset (5–7). Although genetic factors, such as BRCA mutations, and hormonal influences are recognized as major risk contributors, there remains a significant lack of sensitive early detection biomarkers and accurate prognostic stratification tools in clinical practice (8, 9). Therefore, there is an urgent need to further elucidate the underlying pathogenesis of OC to identify novel therapeutic targets.

The underlying cause of this challenge largely stems from the highly heterogeneous and immunosuppressive tumor microenvironment (TME) of OC (10). Unlike most solid tumors, the OC TME is characterized by a unique “ascites circulation” system, forming a dynamic ecosystem composed of tumor cells, immune cells (such as tumor-associated macrophages and T cells), cancer-associated fibroblasts, and a complex extracellular matrix (11–13). Within TME, immunosuppressive mechanisms predominate. Regulatory T cells and M2-polarized macrophages secrete transforming growth factor-β (TGF-β) and interleukin-10 (IL-10), which not only suppress the cytotoxic function of effector T cells but also facilitate tumor invasion and metastasis (14–16). Meanwhile, aberrant expression of immune checkpoint molecules such as PD-1/PD-L1 further enables tumor cells to evade immune surveillance (17). In addition, enhanced angiogenesis and extracellular matrix remodeling within the TME provide a favorable niche for rapid tumor growth and progression, which not only contributes to the high invasiveness and therapeutic resistance of OC but also renders single-target treatment strategies largely ineffective (12, 18, 19). Therefore, a deeper understanding of the cellular and molecular interactions within the OC TME is critical for overcoming diagnostic and therapeutic bottlenecks, identifying novel prognostic biomarkers, and developing effective immunotherapy-based combination strategies.

Among the key immunoregulatory molecules shaping the TME, leukocyte immunoglobulin-like receptor B4 (LILRB4) has recently emerged as an important immune checkpoint receptor involved in tumor immune escape (20). LILRB4 belongs to the LILR family, a subgroup of immunoglobulin superfamily receptors that are predominantly expressed on immune cells and play critical roles in maintaining immune homeostasis by regulating activating and inhibitory signaling thresholds (21, 22). Members of the LILRB subfamily are characterized by immunoreceptor tyrosine-based inhibitory motifs (ITIMs) in their cytoplasmic domains, which transmit inhibitory signals upon ligand engagement and thereby dampen immune activation (23). LILRB4 is mainly expressed on myeloid lineage cells, including monocytes, macrophages, and dendritic cells, which functions as a potent inhibitory receptor that suppresses immune responses through ITIM-mediated signaling pathways (24, 25). In the context of cancer, accumulating evidence suggests that LILRB4 contributes to the establishment of an immunosuppressive TME by regulating myeloid cell differentiation, promoting tumor-associated macrophage polarization toward an M2-like phenotype, and impairing T-cell–mediated anti-tumor immunity (26–28). Moreover, elevated LILRB4 expression has been associated with tumor progression and poor prognosis across multiple malignancies, highlighting its potential as both a prognostic biomarker and a therapeutic target (24, 28–30). However, its precise role and regulatory mechanisms in OC remain insufficiently understood, warranting further investigation.

In this study, we identified LILRB4 as the most prominently expressed member of the LILRB family in OC, showing significantly higher levels in tumor tissues compared with normal ovarian tissues, and its increased expression was closely associated with poor patient prognosis. *In vitro* functional assays revealed that LILRB4 knockdown markedly suppressed OC cell proliferation, migration, and invasion, whereas ectopic overexpression exerted opposite effects and enhanced these malignant behaviors. Further analyses demonstrated that LILRB4 expression positively correlated with tumor-associated macrophage infiltration within the TME and facilitated their polarization toward an immunosuppressive M2c-like phenotype. Mechanistically, our *in vitro* findings indicated that LILRB4 promotes CCL5 secretion from tumor cells, activates the NF-κB p65 signaling pathway, and consequently drives M2c macrophage polarization, thereby contributing to tumor progression and immune suppression. Importantly, inhibition of LILRB4 improved the responsiveness of OC to immunotherapy, highlighting its potential as a therapeutic target in OC.

## 2 Materials and methods

### 2.1 Clinical specimens

OC tissue samples and corresponding normal ovarian tissues were obtained from patients undergoing surgical resection at Shanghai First Maternity and Infant Hospital. A total of 61 OC tissue samples and 20 normal ovarian tissue samples were collected for this study. The inclusion criteria for OC patients in this study were as follows: (1) patients who were pathologically diagnosed with OC; (2) patients who underwent primary surgical treatment at the Shanghai First Maternity and Infant Hospital; and (3) availability of complete clinicopathological information and corresponding tumor tissue samples. The exclusion criteria were as follows: (1) patients who received preoperative chemotherapy or radiotherapy; (2) patients with insufficient or degraded tissue samples unsuitable for analysis; and (3) patients with incomplete clinical or pathological data. The clinical and pathological diagnoses were independently confirmed by at least two experienced pathologists. The study was approved by the Institutional Ethics Committee of the hospital (approval number: GKS2628), and written informed consent was obtained from all participants. All procedures were conducted in accordance with the principles of the Declaration of Helsinki and the International Ethical Guidelines for Health-Related Research Involving Humans issued by the Council for International Organizations of Medical Sciences (CIOMS).

### 2.2 Data acquisition and processing

Gene expression profiles of ovarian cancer (OV) cohorts were obtained from The Cancer Genome Atlas (TCGA, https://portal.gdc.cancer.gov/) database and processed with standard normalization procedures to calculate gene expression levels. The expression of target genes in tumor samples was subsequently extracted and analyzed. For LILRB4, to evaluate its differential expression between tumor and normal tissues, TCGA tumor data were integrated with normal ovarian tissue expression data from the Genotype-Tissue Expression (GTEx) database. Correlation analyses between genes were performed using Spearman correlation tests to assess the associations between LILRB4 and other immune-related genes or biomarkers. All statistical analyses were conducted using R software, and the results were visualized accordingly.

### 2.3 Bioinformatics analysis

Microarray datasets were obtained from the Gene Expression Omnibus (GEO, https://www.ncbi.nlm.nih.gov/geo/) database. Kaplan–Meier survival analysis was conducted using the KM Plotter tool (http://kmplot.com/analysis/) to evaluate the prognostic significance of relevant indicators. The LinkedOmics database (http://www.linkedomics.org) was employed for co-expression analysis and functional enrichment. Additionally, the single-sample Gene Set Enrichment Analysis (ssGSEA) algorithm was applied to estimate the level of immune cell infiltration.

### 2.4 Quantitative real-time polymerase chain reaction (qRT-PCR)

As described in previous studies (31), total RNA was extracted from tissues and cell lines using TRIzol reagent (Takara, Japan). Subsequently, cDNA was synthesized using the PrimeScript RT Master Mix kit (RK20429, ABclonal, China), and quantitative real-time PCR was performed using the RK21203 kit (ABclonal, China). GAPDH or β-actin served as the internal control, and relative gene expression levels were calculated using the 2^−ΔΔCt^ method. The primer sequences are listed in **Supplementary Table 1**.

### 2.5 Western blotting (WB)

As described in previous studies (32), after the tissues and cells are lysed, the supernatant is collected by centrifugation and the total protein concentration is determined. Equal amounts of protein are mixed with loading buffer, denatured at 95 °C for 5 minutes, and separated by sodium dodecyl sulfate-polyacrylamide gel electrophoresis. The protein is transferred onto a polyvinylidene fluoride (PVDF) membrane, and then the membrane is blocked at room temperature with 5% non-fat milk. Afterwards, the membrane is incubated with the primary antibody at 4 °C overnight, and then incubated with the horseradish peroxidase (HRP)-conjugated secondary antibody at room temperature for 1 hour. The protein bands are detected using an enhanced chemiluminescence kit and visualized using a biological imaging system.

### 2.6 Antibodies and reagents

All antibodies used in this study were commercially available: LILRB4 (A7073; ABclonal, Wuhan, China; 1:2000 for WB and 1:200 for immunohistochemistry), glyceraldehyde-3-phosphate dehydrogenase (GAPDH) (AB2100; NCM Biotech, Suzhou, China; 1:20,000), F4/80 (A27257; ABclonal, Wuhan, China; 1:200), Ki67 (A20018; ABclonal, Wuhan, China; 1:200), CD68 (A24386PM; ABclonal, Wuhan, China; 1:200), phospho-NF-κB (p-NF-κB; TP56372; Abmart, Shanghai, China; 1:1000), CD163 (A8383SP; ABclonal, Wuhan, China; 1:200), CD8 (A23081; ABclonal, Wuhan, China; 1:200), granzyme B (GZMB) (A2557; ABclonal, Wuhan, China; 1:200), and goat anti-rabbit IgG (H+L)-HRP conjugate (LF102; Epizyme Biotech, Shanghai, China; 1:3000). All antibodies were diluted according to the manufacturers’ instructions. Detailed information on antibodies and reagents used for flow cytometry is provided in **Supplementary Table 2**.

### 2.7 Immunohistochemistry (IHC)

Tissue sections were dewaxed in xylene and rehydrated through a graded ethanol series. Endogenous peroxidase activity was blocked with 3% hydrogen peroxide, followed by heat-induced antigen retrieval. Sections were incubated with primary antibodies overnight at 4 °C and then with the corresponding secondary antibodies for 1 h at room temperature. Immunoreactivity was visualized using DAB, and the slides were counterstained with hematoxylin and mounted.

### 2.8 ROC curve analysis

In the TCGA-OV cohort, transcriptomic data and corresponding clinical information were obtained, and LILRB4 expression levels were extracted. Based on patient survival outcomes, receiver operating characteristic (ROC) curve analysis was performed using the R package “pROC” to evaluate the predictive performance of LILRB4 expression. The area under the curve (AUC) was calculated to assess discriminatory ability, and ROC curves were generated for visualization.

### 2.9 Cell culture and stable cell line construction

Human cell lines IOSE80 (normal ovarian epithelial cells), HEY, A2780, CAOV3, OVCAR8, and ES2 (OC cell lines), along with THP-1 and 293T cells, as well as the murine EOC cell line ID8, were obtained from our laboratory and maintained following previously reported protocols (10, 33). THP-1 cells were cultured in Roswell Park Memorial Institute (RPMI) 1640 medium, while the remaining cell lines were grown in Dulbecco’s Modified Eagle Medium (DMEM) (both from Wuhan Procell Biotechnology Co., Ltd, Wuhan, China), all supplemented with 10% fetal bovine serum (FBS; C9050, NCM Biotech, Suzhou, China) and 1% penicillin/streptomycin (Gibco, Grand Island, NY, USA). Stable LILRB4 knockdown cell lines were established as previously described (34). The corresponding plasmids and negative control (NC) vectors were designed and synthesized by YouBio (Changsha, China).

### 2.10 Cell counting kit-8 assay

CCK-8 reagent (C6005, NCM Biotech, Suzhou, China) was used to evaluate the proliferation ability of OC cells. Cells were seeded in 96-well plates at 1 × 10^3^ cells per well. After culturing for 0, 24, 48, 72, and 96 h, CCK-8 solution (10 µL) was added to each well, incubated for 2 h in an incubator, and then a spectrophotometer was used to evaluate the absorbance at 450 nm.

### 2.11 Transwell assays

As described previously (35), a Transwell assay was performed on tumor cells. After crystal violet staining, images were captured using a microscope, and the number of cells was subsequently quantified using ImageJ software.

### 2.12 Animal experiment

All *in vivo* experiments were conducted in accordance with the National Institutes of Health Guide for the Care and Use of Laboratory Animals and were approved by the Animal Care Committee of Tongji University (approval number: TJBG30326101). For the orthotopic tumor model, 6-week-old female C57BL/6 mice were used for tumor implantation. A total of 1 × 10^6^ ID8 cells (20 μL; control group and sh-LILRB4 group) were mixed at a 1:1 ratio with Matrigel (20 μL, D23016-0010, D1 Medical Technology, Hangzhou, China) and injected into the ovarian bursa to establish the orthotopic tumor model. Tumor growth was monitored, and 40 days after injection, mice were anesthetized with isoflurane and euthanized by cervical dislocation. Tumors were then collected for further analysis. For the *in vivo* treatment model, mice received intraperitoneal injections of 200 μg anti-PD-1 antibody (S0B0594, STARTER) or isotype control antibody on days 10, 15, and 25 after orthotopic implantation, with mice randomly assigned to each group. 40 days after implantation, mice were anesthetized with isoflurane, euthanized by cervical dislocation, and tumors were harvested for further analysis.

### 2.13 Flow cytometry

Consistent with previous studies (34), cells were harvested, washed twice with phosphate-buffered saline (PBS), and resuspended in staining buffer. Single-cell suspensions were incubated with the indicated fluorochrome-conjugated antibodies for 30 min at 4 °C in the dark. After staining, cells were washed twice with PBS and analyzed using a FACSCalibur flow cytometer (BD Biosciences, New Jersey, USA). For macrophage phenotyping, differentiated THP-1-derived macrophages were first identified based on forward scatter (FSC) and side scatter (SSC) characteristics to exclude debris, followed by doublet exclusion using FSC-H versus FSC-A gating. M1 macrophages were defined as CD11b^+^CD86^+^ cells, whereas M2c-like macrophages were defined as CD11b^+^CD163^+^ cells. Fluorescence compensation was performed using single-stained controls according to the manufacturer’s recommendations. Corresponding isotype controls were included to assist gate setting and evaluation of nonspecific staining. Data acquisition and analysis were performed using FlowJo software (V10). All flow cytometry experiments were independently repeated at least three times, and the number of biological replicates for each experiment is indicated in the corresponding figure legends.

### 2.14 Enzyme-linked immunosorbent assay

Secretion of CCL5 into the culture supernatant from cells was detected using ELISA kits (SYP-H4234, UpingBio, Hangzhou, China) according to the manufacturer’s instructions. The ELISA kit has a sensitivity of less than 10 pg/mL and a detection range of 62.5–2000 pg/mL. For each assay, 50 μL of culture supernatant was used for measurement.

### 2.15 LILRB4 expression in OC immune cells

Single-cell RNA sequencing data were analyzed using the Tumor Immune Single-cell Hub 2 (TISCH2, https://tisch.compbio.cn/home/) online platform to evaluate the expression of LILRB4 in OC datasets. The expression levels of LILRB4 were systematically assessed across different immune cell populations within the TME to characterize its cell type–specific distribution.

### 2.16 Immunotherapy cohort analysis

The association between LILRB4 expression and clinical outcomes following immunotherapy was evaluated using the immunotherapy module of the Kaplan–Meier Plotter database. Patients with esophageal adenocarcinoma who received immune checkpoint inhibitor-based treatment were included in the analysis. Tumors were stratified into high- and low-LILRB4 expression groups according to the optimal cutoff automatically determined by the Kaplan–Meier Plotter platform. Overall survival (OS) and progression-free survival (PFS) were analyzed using Kaplan–Meier survival curves, and differences between groups were assessed using the log-rank test. Hazard ratios (HR) and corresponding 95% confidence intervals were obtained from the database. Given the limited availability of publicly accessible immunotherapy cohorts with both gene expression and clinical outcome data, these analyses were intended to provide exploratory evidence regarding the potential association between LILRB4 expression and immunotherapy response.

### 2.17 Jurkat T cell activation assay

For activation, Jurkat cells were seeded into 12-well plates at a density of 1 × 10^6^ cells per well and stimulated with 25 μL of ImmunoCult™ Human CD3/CD28 T Cell Activator (STEMCELL, Canada) for 24 hours. After stimulation, the cells were co-cultured with CM from macrophages for subsequent analysis. Expression of activation markers such as CD69 was evaluated by flow cytometry.

### 2.18 Statistical analysis

Statistical analyses were performed using R (v4.2.2) or GraphPad Prism (v9). All experiments were performed with at least three independent biological replicates (n ≥ 3), and the exact sample size for each experiment is indicated in the corresponding figure legends. Cell counts were quantified using ImageJ, and flow cytometry data were analyzed using FlowJo (v10). For comparisons between two groups, statistical significance was assessed using Student’s t-test (two-tailed), and for multiple group comparisons, one-way ANOVA followed by appropriate *post hoc* tests was applied as indicated in the figure legends. Data distribution was assessed prior to the application of parametric tests, and analyses were performed under the assumption of normal distribution. A p-value < 0.05 was considered statistically significant.

## 3 Results

### 3.1 LILRB4 upregulation in OC is associated with poor prognosis

**Figure 1:**
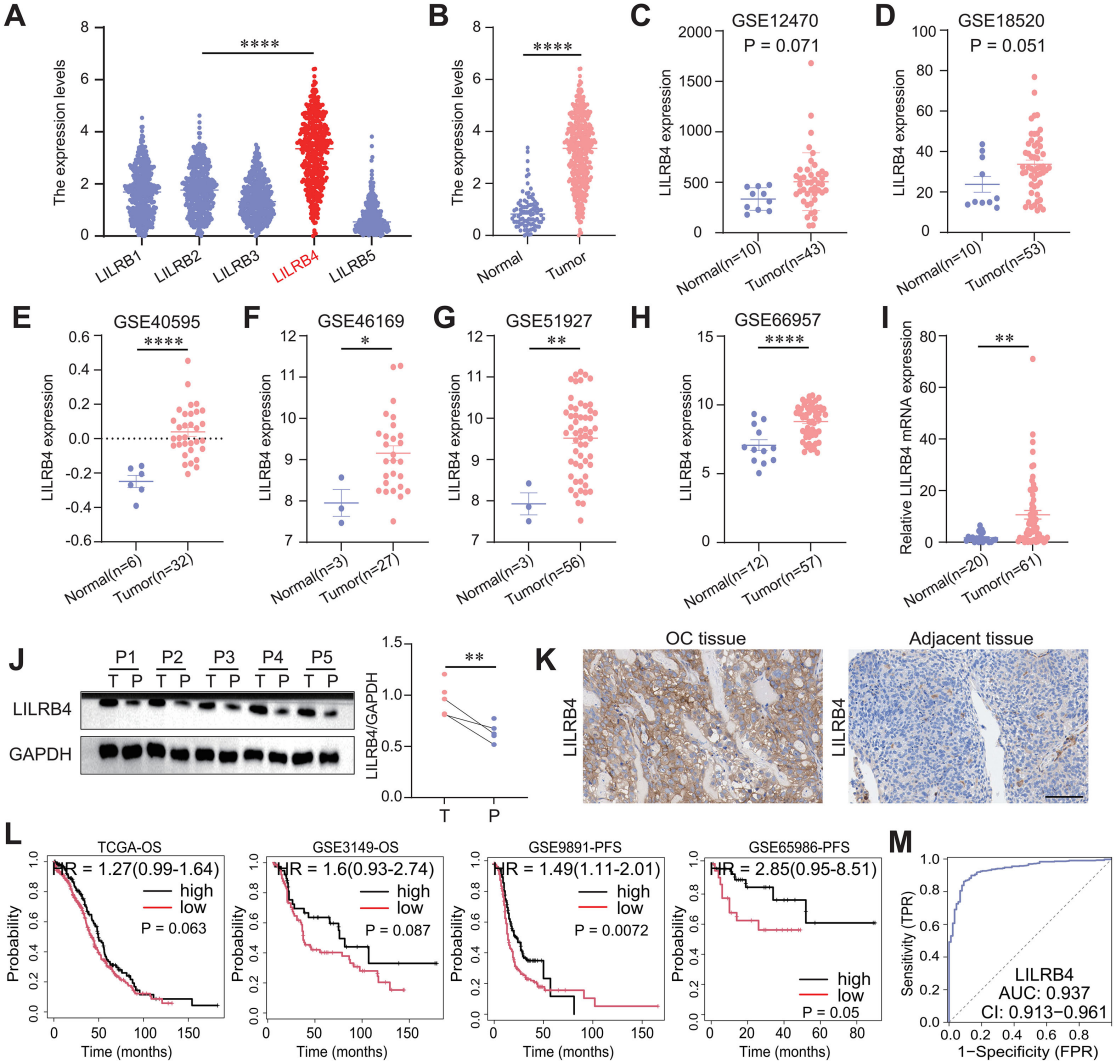
Elevated LILRB4 expression predicts poor prognosis in OC. **(A)** Expression profiles of LILRB family genes in OC cohort from TCGA database. **(B)** Comparison of LILRB4 mRNA expression levels between OC and normal tissues in the TCGA dataset combined with GTEx database. **(C–H)** Validation of LILRB4 mRNA expression differences between OC and normal tissues across 6 independent GEO datasets (GSE12470, GSE18520, GSE40595, GSE46169, GSE51927, and GSE66957). **(I)** Further validation of LILRB4 expression in human OC and normal tissues collected from Shanghai First Maternity and Infant Hospital. **(J)** Protein expression levels of LILRB4 in OC tissues **(T)** and adjacent non-tumor tissues **(P)** were assessed by WB (left). Densitometric quantification was performed using ImageJ software and normalized to GAPDH expression (n = 5) (right). **(K)** IHC staining showing LILRB4 expression in tumor and adjacent non-tumor tissues. Scale bar = 200 μm. **(L)** Kaplan–Meier survival analysis based on TCGA and GEO datasets evaluating the association between LILRB4 expression and OS and PFS. Statistical significance was determined using the log-rank test. **(M)** ROC curve analysis showing the diagnostic performance of LILRB4, with the AUC indicating predictive accuracy. Data are presented as mean ± standard deviation (SD). Statistical significance was determined using an unpaired two-tailed Student’s t-test. *p < 0.05, **p < 0.01, ****p < 0.0001.

We initially analyzed the copy number variations (CNVs) of LILRB family genes using the cBioPortal database. Among the 5 molecules analyzed, we found that they had different proportions of CNVs (**Supplementary Figure 1**), suggesting that changes in their expression levels might affect the progression of OC. Subsequently, we further investigated the expression of LILRB family genes in human OC using the TCGA dataset. We found that LILRB4 had the highest expression level among these genes in OC (**Figure 1A**). Therefore, we chose LILRB4 as the focus of our subsequent research. We then overlaid the GTEx database to analyze the expression differences of LILRB4. We observed that LILRB4 was more significantly expressed in OC samples compared to normal samples (**Figure 1B**). We further utilized the GEO public dataset to study the expression of LILRB4, and the results further indicated that the expression level of LILRB4 was upregulated in human OC tissues (**Figures 1C–H**). Next, we evaluated the expression of LILRB4 in human OC and normal tissues obtained from our hospital. Consistent with the results, the expression level of LILRB4 was significantly higher in OC tissues and positively correlated with higher tumor grade, advanced FIGO stage, and larger tumor size (**Figure 1I**; **Table 1**). The WB results also confirmed that the protein level of LILRB4 was higher in cancer tissues compared to the matched adjacent tissues (**Figure 1J**). Additionally, IHC analysis showed that the expression of LILRB4 was significantly increased in cancer tissues compared to adjacent tissues (**Figure 1K**).

**Table 1:** Relationship between LILRB4 mRNA expression and clinicopathological parameters in OC patients.

Characteristics	Low expression of LILRB4	High expression of LILRB4	P value
n	27	34	
Age (years)			0.640
≥ 50	19	22	
< 50	8	12	
Grade			0.046
G1+G2	12	7	
G3	15	27	
FIGO-Stage			0.067
I+II	19	16	
III+IV	8	18	
Histopathological type			0.736
High-grade serous carcinoma	17	25	
Endometrioid carcinoma	2	3	
Clear cell carcinoma	7	5	
Mucinous carcinoma	1	1	
Tumor size(cm)			0.039
< 8.5	15	10	
≥ 8.5	12	24	
CA125(U/mL)			0.702
≥ 500	6	9	
< 500	21	25	

Chi-square test.

Finally, Kaplan-Meier survival analysis of multiple cohorts indicated that patients with higher LILRB4 levels in OC had poorer OS and PFS (**Figure 1L**). Subsequent ROC curve analysis determined that LILRB4 was an independent prognostic factor for OC patients (**Figure 1M**). These findings suggest that LILRB4 is upregulated in OC, and its downregulation may serve as a marker of a favorable prognosis.

### 3.2 LILRB4 enhances malignant phenotypes of OC *in vitro* and *in vivo*

**Figure 2:**
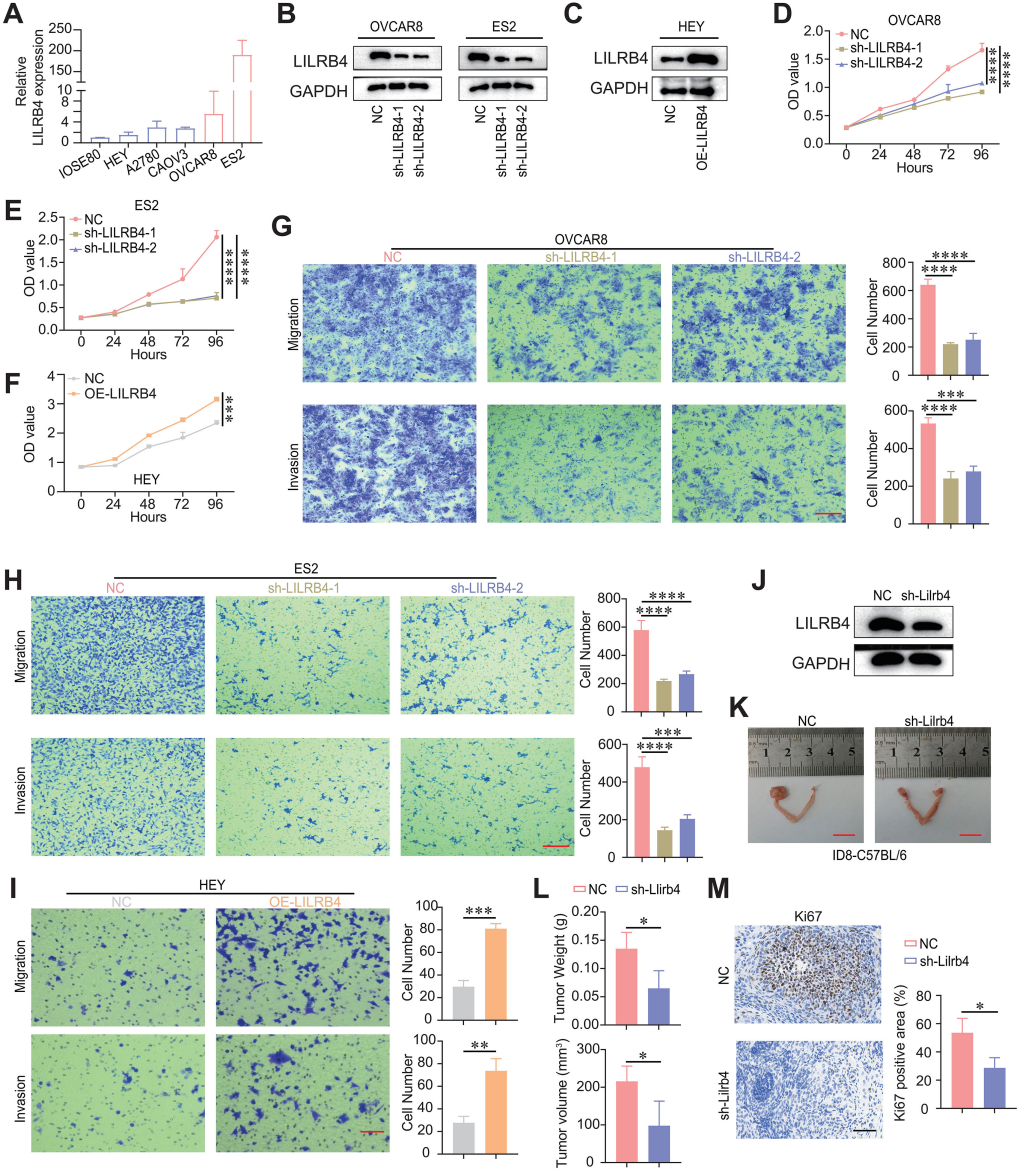
Downregulation of LILRB4 suppresses the malignant phenotypes of OC cells. **(A)** mRNA expression levels of LILRB4 in the normal ovarian epithelial cell line IOSE80 and multiple OC cell lines (HEY, A2780, CAOV3, OVCAR8, and ES2). **(B)** WB analysis confirming the protein knockdown efficiency of LILRB4 in OVCAR8 (left) and ES2 (right) cells; representative images are shown. **(C)** WB analysis confirming the protein overexpression efficiency of LILRB4 in HEY; representative images are shown. **(D, E)** Cell proliferation of OVCAR8 **(D)** and ES2 **(E)** cells in the NC and LILRB4 knockdown groups, as assessed by CCK-8 assay. **(F)** Proliferation of HEY cells with NC or LILRB4 overexpression, evaluated by CCK-8 assay. **(G–I)** Transwell assays demonstrating the migration (up) and invasion (down) abilities of OVCAR8 **(G)**, ES2 **(H)** and HEY **(I)** cells under the indicated treatments. All images are shown at the same magnification with a scale bar of 100 μm. **(J)** WB analysis confirming the protein knockdown efficiency of Lilrb4 in the ID8 cell line; representative images are shown. **(K)** Representative images of orthotopic tumors in C57BL/6 mice (n = 4 per group) implanted with ID8 cells (NC vs. sh-Lilrb4), with a scale bar of 10 mm. **(L)** Comparison of tumor weight (up) and tumor volume (down) between the two groups shown in **(K)**. **(M)** IHC analysis of Ki67 expression in orthotopic tumor tissues, including representative images (left) and quantification of Ki67-positive areas (right) (n = 3). All panels are shown at the same magnification with a scale bar of 100 μm. Data are presented as mean ± SD. Statistical analysis was performed using one-way ANOVA followed by Tukey’s multiple comparisons test (D, E, G, H), or unpaired two-tailed Student’s t-test (F, I, L, M). *p < 0.05, **p < 0.01, ***p < 0.001, ****p < 0.0001.

Next, we investigated the role of LILRB4 in the malignant phenotype of OC. Its effects on cellular behavior were evaluated both *in vitro* and *in vivo*. We first assessed LILRB4 mRNA expression in tumor cell lines. Relatively high expression was observed in OVCAR8 and ES2 cells, while HEY cells showed relatively low expression (**Figure 2A**). Based on this, stable LILRB4 knockdown cell lines were established in OVCAR8 and ES2, and knockdown efficiency was confirmed by WB (**Figure 2B**). Additionally, stable LILRB4 overexpression cell lines were established in HEY cells (**Figure 2C**). To evaluate the impact of LILRB4 on cell proliferation, a CCK-8 assay was performed, demonstrating that LILRB4 knockdown significantly reduced the proliferative capacity of OVCAR8 and ES2 cells, while LILRB4 overexpression in HEY cells enhanced proliferation compared with control cells (**Figures 2D–F**). Transwell migration assays showed that the migratory ability of LILRB4-deficient cells was markedly decreased, whereas LILRB4-overexpressing HEY cells exhibited increased migration (**Figures 2G–I**, up). Similarly, invasion assays revealed reduced invasive capacity following LILRB4 knockdown, and increased invasive capacity upon LILRB4 overexpression, indicating that LILRB4 promotes invasion *in vitro* (**Figures 2G–I**, down).

To further investigate its role *in vivo*, a stable LILRB4 knockdown ID8 cell line was generated and orthotopically implanted into the ovaries of mice (**Figures 2J, K**). The results showed that LILRB4 deficiency suppressed tumor growth, as evidenced by reduced tumor volume and weight (**Figure 2L**). Consistently, Ki67 expression in tumor tissues was also decreased (**Figure 2M**). Overall, these findings suggest that downregulation of LILRB4 attenuates the tumorigenic potential of OC cells both *in vitro* and *in vivo*.

### 3.3 LILRB4 promotes macrophage infiltration in the OC microenvironment

To further explore the potential role of LILRB4 in OC, we performed a co-expression analysis using the LinkedOmics database. Spearman’s correlation analysis identified genes significantly associated with LILRB4 (**Supplementary Figure 2A**), and the top 50 positively and negatively correlated genes were visualized in heatmaps (**Supplementary Figures 2B, C**). KEGG pathway analysis revealed that these genes were enriched in pathways such as Th17 cell differentiation, natural killer cell–mediated cytotoxicity, and Th1/Th2 cell differentiation (**Supplementary Figure 2D**), suggesting a potential role for LILRB4 in shaping the tumor immune microenvironment (TIME) and promoting tumor progression.

**Figure 3:**
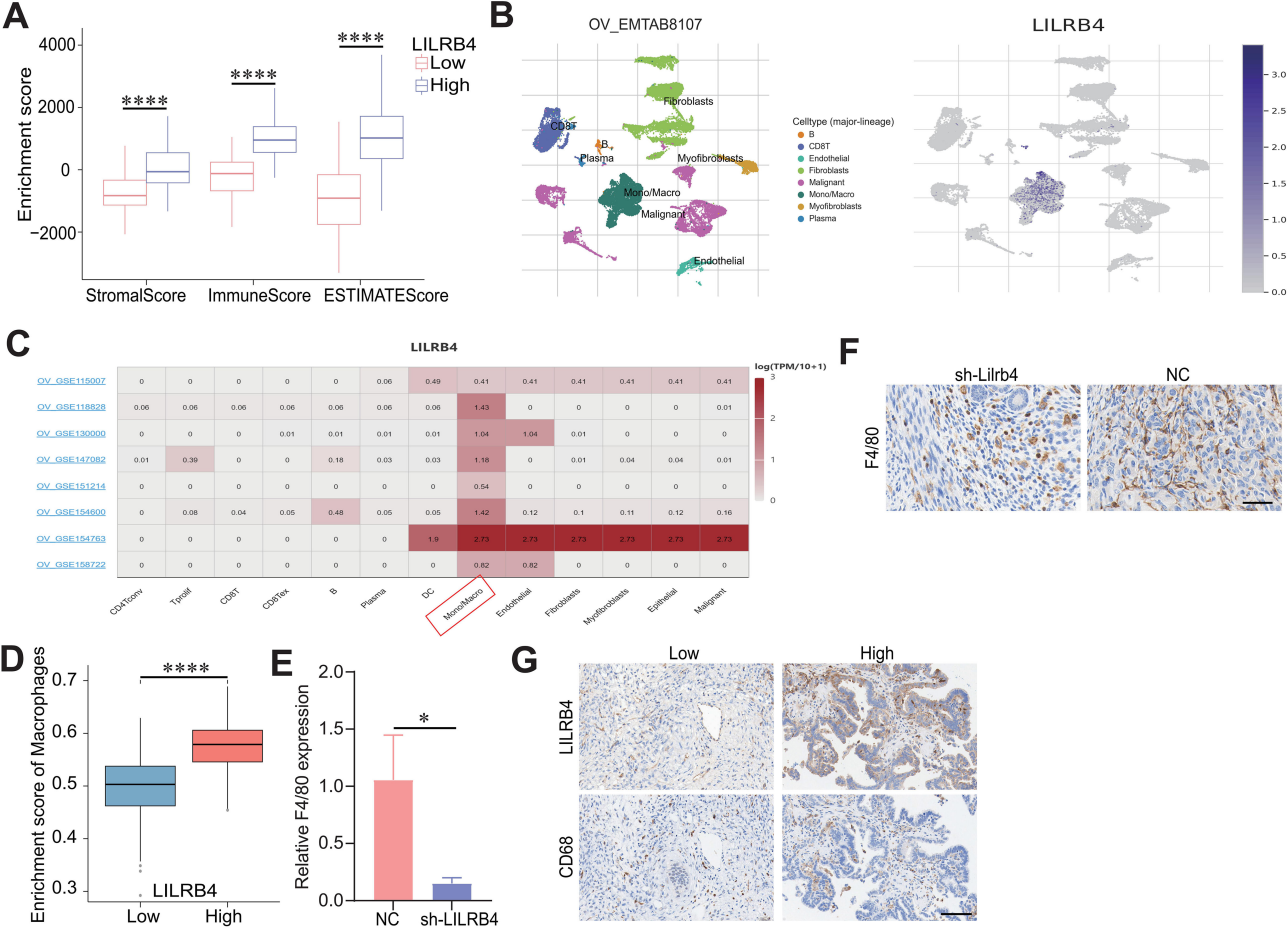
LILRB4 is associated with macrophages in the OC immune microenvironment. **(A)** Correlation of StromalScore, ImmuneScore, and ESTIMATEScore with LILRB4 expression in TCGA-OV patients. **(B)** UMAP visualization of single-cell RNA-seq data from the OV_EMTAB8107 tumor dataset (left) and corresponding LILRB4 expression across different cell clusters (right). **(C)** Distribution of LILRB4 expression across various cell types in GEO-derived single-cell datasets, analyzed using the TISCH2 database (http://tisch.comp-genomics.org/), with darker colors indicating higher expression levels. **(D)** Correlation between macrophage enrichment scores and LILRB4 expression in TCGA-OV samples, as determined by ssGSEA. **(E)** Relative mRNA expression of F4/80 in orthotopic ovarian tumor models (NC and sh-LILRB4 groups). **(F)** Representative IHC images showing F4/80 expression in orthotopic ovarian tumors (n = 3). Scale bar, 100 μm. **(G)** Representative IHC staining of CD68 and LILRB4 in corresponding regions of human OC tissues (n = 3). All panels are shown at the same magnification. Scale bar, 100 μm. Data are presented as mean ± SD. Statistical analysis was performed using unpaired two-tailed Student’s t-test. *p < 0.05, ****p < 0.0001.

To test our hypothesis, we first applied the ESTIMATE algorithm to the TCGA-OV cohort and observed that tumors with elevated LILRB4 expression exhibited higher immune scores (**Figure 3A**). We next analyzed a single-cell RNA-seq dataset of OC (OV_EMTAB8107), which revealed that LILRB4 expression was predominantly enriched in the monocyte/macrophage (Mono/Macro) population among the major cell types (**Figure 3B**). This observation was further validated in additional single-cell datasets (**Figure 3C**), collectively suggesting a close association between LILRB4 and macrophages within the TME. Furthermore, ssGSEA demonstrated that increased LILRB4 expression in TCGA-OV samples was accompanied by higher macrophage enrichment scores (**Figure 3D**). Consistent with this finding, correlation analyses showed that LILRB4 expression was significantly positively associated with both macrophage infiltration and the tumor-associated macrophage marker CD68 (**Supplementary Figure 3A**). To further validate these, we examined previously established orthotopic mouse tumor models. qRT-PCR and IHC analyses confirmed differential macrophage infiltration between the two groups, with tumors expressing higher levels of LILRB4 exhibiting increased expression of the macrophage marker F4/80 (**Figures 3E, F**). Similarly, in human OC tissues, elevated LILRB4 expression was closely associated with enhanced macrophage infiltration (**Figure 3G**). Taken together, these findings indicate that LILRB4 may contribute to the remodeling of the immune microenvironment in OC by promoting macrophage infiltration.

### 3.4 LILRB4 drives macrophage polarization toward an M2c-like phenotype

**Figure 4:**
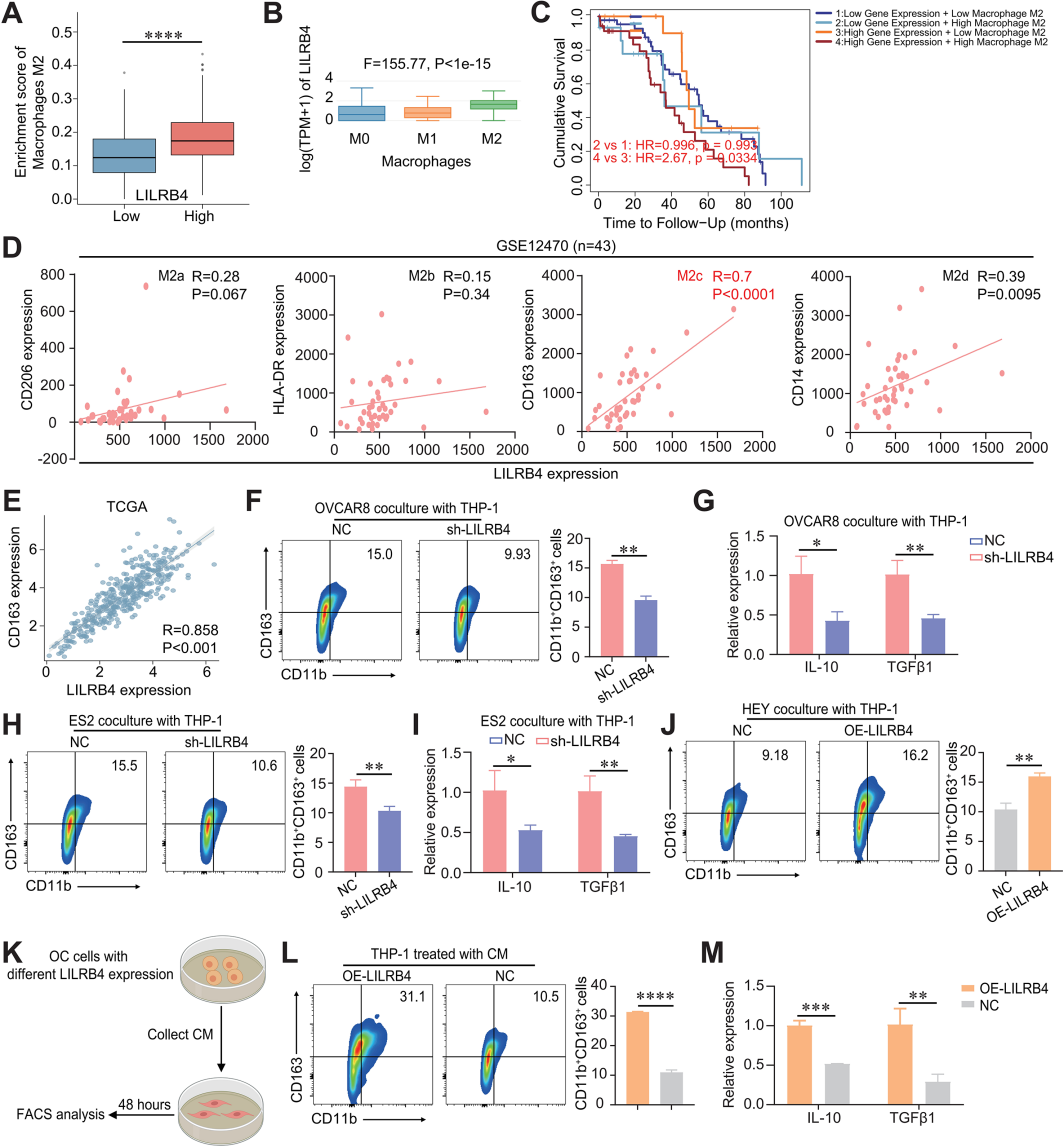
LILRB4 regulates M2c macrophage polarization. **(A)** Patients in the TCGA-OV cohort were stratified into high- and low-LILRB4 expression groups to assess differences in M2 macrophage infiltration. **(B)** LILRB4 expression levels in M0, M1, and M2 macrophages based on the GEPIA database. **(C)** TCGA-OV samples were categorized into high and low M2 macrophage infiltration groups. OS was analyzed based on LILRB4 expression using the TIMER database; statistical significance was determined by the log-rank test. **(D)** Correlation analysis LILRB4 and CD206 (M2a), HLA-DR (M2b), CD163 (M2c), CD14 (M2d) by GSE12470 cohort. **(E)** Correlation analysis between LILRB4 expression and the M2c markers CD163 in the TCGA-OV cohort. **(F, H)** Flow cytometry analysis of CD163 expression in macrophages following co-culture with OVCAR8 **(F)** or ES2 **(H)** cells with NC or LILRB4 knockdown. **(G, I)** qRT-PCR analysis of IL-10 and TGFβ1 mRNA expression in macrophages co-cultured with OVCAR8 **(G)** or ES2 **(I)** cells under control (NC) or LILRB4 knockdown conditions. **(J)** Flow cytometry analysis of CD163 expression in macrophages following co-culture with HEY cells with NC or OE-LILRB4. **(K)** A schematic diagram showing the process of collecting tumor cell CM and co-culturing it with macrophages for 48 hours before conducting flow cytometry detection. **(L)** Macrophages were treated with CM from LILRB4-overexpressing tumor cells or control tumor cells, and the proportion of CD163-positive macrophages was assessed (left) and quantitatively compared between the 2 groups (right). **(M)** Macrophages were treated with CM from LILRB4-overexpressing tumor cells or control tumor cells, and the expression levels of IL-10 and TGFβ1 in macrophages were measured by qRT-PCR. Data are presented as mean ± SD. Statistical analysis was performed using unpaired two-tailed Student’s t-test (A, F-J, L, M). *p < 0.05, **p < 0.01, ***p < 0.001, ****p < 0.0001.

Based on the observation that LILRB4 promotes macrophage infiltration into the TME, we next investigated whether it also influences macrophage polarization. Tumor-associated macrophages exhibit considerable plasticity and are broadly categorized into classically activated M1-like macrophages, which exert anti-tumor effects through pro-inflammatory and immunostimulatory functions, and alternatively activated M2-like macrophages, which are generally associated with tumor progression (36–38). Since LILRB4 is similar to M2 macrophages in its prognosis for OC, we hypothesized that LILRB4 may preferentially drive M2 polarization, thereby contributing to immunosuppression. Consistent with this hypothesis, ssGSEA analysis of TCGA-OV samples demonstrated that elevated LILRB4 expression was accompanied by increased enrichment of M2 macrophages (**Figure 4A**). Moreover, LILRB4 expression was significantly higher in M2 macrophages compared with M0 and M1 subsets (**Figure 4B**). Notably, Survival analysis further revealed that patients with both high LILRB4 expression and increased M2 macrophage infiltration exhibited the poorest prognosis, whereas low LILRB4 expression was associated with reduced M2 infiltration (**Figure 4C**). Therefore, we next further investigated the association between LILRB4 expression and different M2 macrophage subtypes, including M2a, M2b, M2c, and M2d (39) and we found that LILRB4 is positively correlated with CD163, a classical marker of M2c (**Figures 4D, E**). Collectively, these preliminary findings suggest that LILRB4 may be significantly associated with M2c macrophage polarization in the OC microenvironment.

To further validate the relationship between tumor-derived LILRB4 and macrophage M2c polarization, we employed a Transwell co-culture system that allows paracrine interactions without direct cell–cell contact (17, 33). Downregulation of LILRB4 in tumor cells led to a marked decrease in the mRNA expression of M2c-associated markers, including IL-10 and TGFβ1 (**Figures 4G, I**; **Supplementary Figure 3B**). Consistently, flow cytometry analysis demonstrated reduced expression of the M2c surface marker CD163 at the protein level (**Figures 4F, H**). In contrast, LILRB4 overexpression significantly increased the expression of CD163 levels, further supporting a promotive role of LILRB4 in M2c polarization (**Figure 4J**). To further clarify that these effects are not solely caused by the direct interaction between tumor cells and macrophages mediated by LILRB4, but rather by broader inflammatory or TME-related changes, we collected conditioned media (CM) from tumor cells and conducted macrophage co-culture experiments. We found that the CM from LILRB4-overexpressing tumor cells significantly increased the expression of M2 macrophage marker CD163, with a significant difference compared to the CM from control cells (**Figures 4K–M**). This supports the view that LILRB4 may at least partially regulate macrophage polarization through soluble factors produced by tumor cells. Overall, LILRB4 promotes M2c macrophage polarization in OC.

### 3.5 LILRB4-induced CCL5 secretion promotes M2c macrophage differentiation in an NF-κB–dependent manner

**Figure 5:**
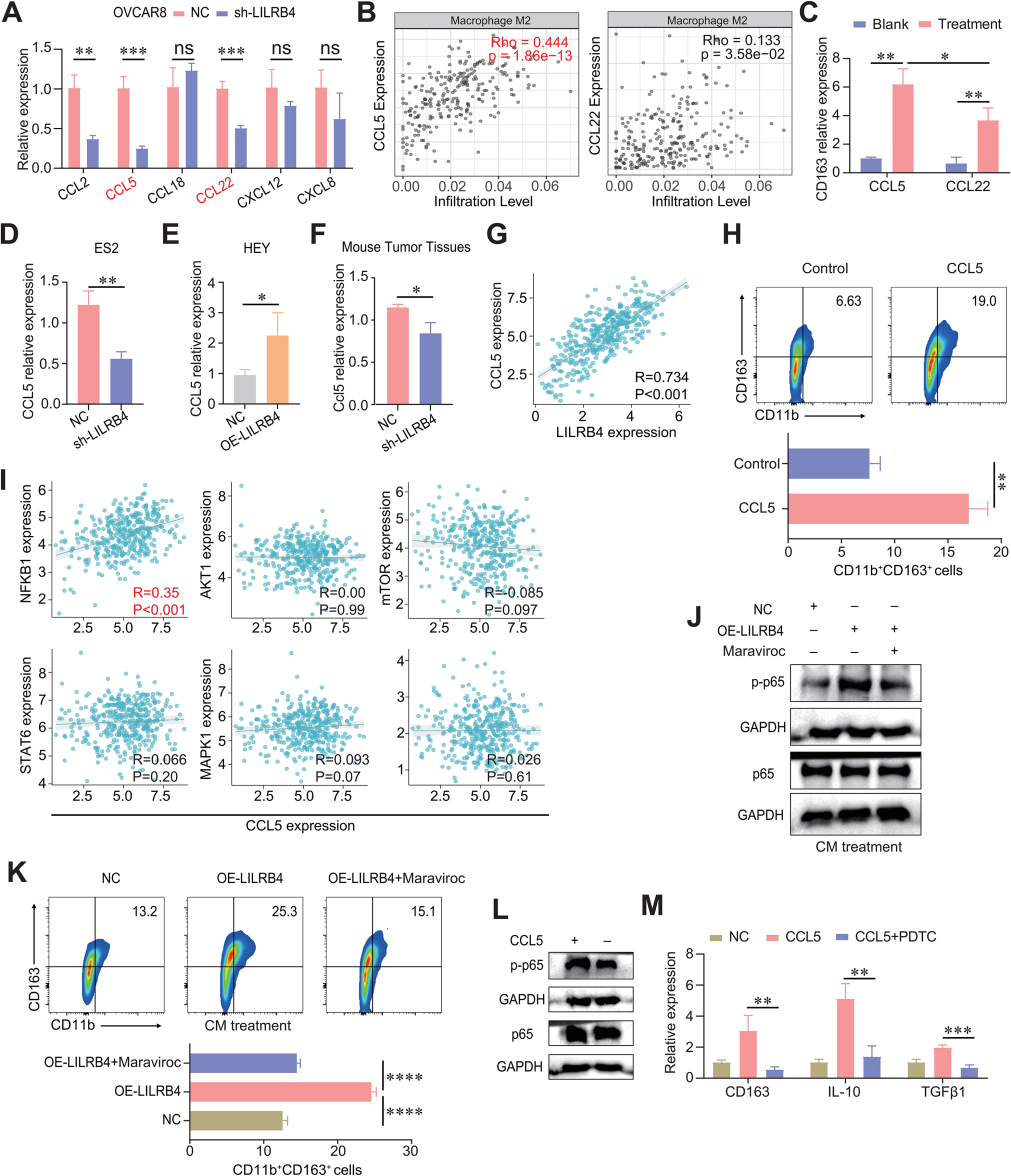
LILRB4 promotes M2c macrophage differentiation via CCL5/NF-κB signaling. **(A)** In the OVCAR8 cells, qRT-PCR was used to analyze the mRNA expression levels of CCL2, CCL5, CCL18, CCL22, CXCL12 and CXCL8. **(B)** Correlation analysis between CCL5 (left) and CCL22 (right) expression and M2 macrophage infiltration based on the TIMER database. **(C)** Expression levels of the M2c macrophage marker CD163 in macrophages following treatment with CCL5 or CCL22. **(D)** The expression of CCL5 mRNA in ES2 cells either under normal control conditions or under sh-LILRB4 treatment was analyzed by qRT-PCR. **(E)** The expression of CCL5 mRNA in HEY cells under different treatment was analyzed by qRT-PCR. **(F)** qRT-PCR analysis of CCL5 mRNA expression in ovarian tumor tissues from the indicated groups. **(G)** Correlation analysis between LILRB4 expression and CCL5 in the TCGA-OV cohort. **(H)** Flow cytometry analysis of CD11b^+^CD163^+^ macrophages in the control and CCL5-treated groups (left), with corresponding quantification shown on the right. **(I)** Correlation analysis between CCL5 expression and key signaling molecules (NFKB1, AKT1, mTOR, STAT6, MAPK1, and NOTCH1) in the TCGA-OV cohort. **(J)** WB analysis showing p-p65 levels in macrophages treated with CM from control or LILRB4-overexpressing tumor cells or maraviroc, indicating activation of the NF-κB signaling pathway. **(K)** Expression levels of CD163 in macrophages under the above treatment conditions (up), with quantitative analysis of the different groups (down). **(L)** Assessment of p-p65 activation in macrophages following CCL5 treatment. **(M)** qRT-PCR analysis of CD163, IL-10, and TGFβ1 mRNA expression in macrophages treated with control (NC) or CCL5 or CCL5 with PDTC. Data are presented as mean ± SD. Statistical analysis was performed with unpaired two-tailed Student’s t-test (A, C-F, H) or unpaired two-tailed Student’s t-test (K, M). *p < 0.05, **p < 0.01, ***p < 0.001, ****p < 0.0001.

Given these findings, we next sought to investigate whether LILRB4 downregulation alters the secretion profile of chemokines in tumor cells, thereby contributing to the impaired M2c polarization (40–42). Specifically, we aimed to determine whether changes in certain soluble factors mediated the reduced M2c-associated phenotype observed following LILRB4 knockdown. Firstly, qRT-PCR was performed to assess the expression of related chemokines in OC cells. The results showed that alterations in LILRB4 expression had the most pronounced effects on CCL5 and CCL22 in cells (**Figure 5A**). Subsequent analysis of public databases revealed that, in OC, CCL5 exhibited a significantly stronger correlation with M2 macrophages than CCL22 (**Figure 5B**). To further compare their functional relevance, additional qRT-PCR analysis was performed. Recombinant CCL22 was found to influence the expression of M2c macrophage markers; however, its effect was consistently weaker than that of CCL5 (**Figure 5C**). Therefore, CCL5 was selected for subsequent mechanistic investigations. Accordingly, CCL5 expression was further validated in ES2 and HEY cells, where LILRB4 knockdown in ES2 cells led to a significant decrease in CCL5 expression, whereas LILRB4 overexpression in HEY cells resulted in a marked increase in CCL5 levels (**Figures 5D, E**), which was additionally confirmed by ELISA (**Supplementary Figure 4**). Consistently, mouse tumor tissue experiments also supported a positive regulatory relationship between LILRB4 and CCL5 in OC (**Figure 5F**). These findings indicate that LILRB4 is closely associated with CCL5 signaling in OC (**Figure 5G**). We then explored the role of CCL5 in M2c macrophage polarization. Flow cytometry analysis demonstrated that exogenous CCL5 significantly increased CD163 expression on macrophages (**Figure 5H**). Overall, LILRB4 promotes M2c macrophage polarization in OC, at least in part through upregulation of CCL5 signaling within the TME.

M2 polarization is typically accompanied by activation of multiple intracellular signaling pathways, including NF-κB, AKT–mTOR, STAT6, MAPK, and Notch (43–45). To elucidate the mechanism underlying LILRB4-induced CCL5-mediated M2c polarization, we examined the association between CCL5 expression and key components of these pathways using the TCGA-OV cohort. Correlation analysis revealed that LILRB4 exhibited the strongest association with NFKB1 compared with other signaling molecules (**Figure 5I**). To further evaluate the effect of the LILRB4–CCL5–p65 axis on M2c macrophage polarization, CM were collected from control and LILRB4-overexpressing tumor cells and used to treat macrophages. Treatment with CM from LILRB4-overexpressing cells significantly increased p65 phosphorylation (p-p65), a key indicator of NF-κB pathway activation (**Figure 5J**). Notably, co-treatment with the FDA-approved CCR5 antagonist maraviroc markedly attenuated CM-induced M2 polarization and reversed p65 phosphorylation levels (**Figures 5J, K**). In addition, WB indicated that CCL5 stimulation activates p65 signaling in macrophages, while pharmacological inhibition of NF-κB using PDTC partially abrogated the promotive effect of CCL5 on M2c macrophage polarization (**Figures 5L, M**). Collectively, these results suggest that LILRB4 promotes macrophage polarization toward an M2c phenotype by enhancing CCL5 secretion and activating NF-κB signaling.

### 3.6 CCL5-mediated M2c macrophages promote tumor progression and immune suppression

**Figure 6:**
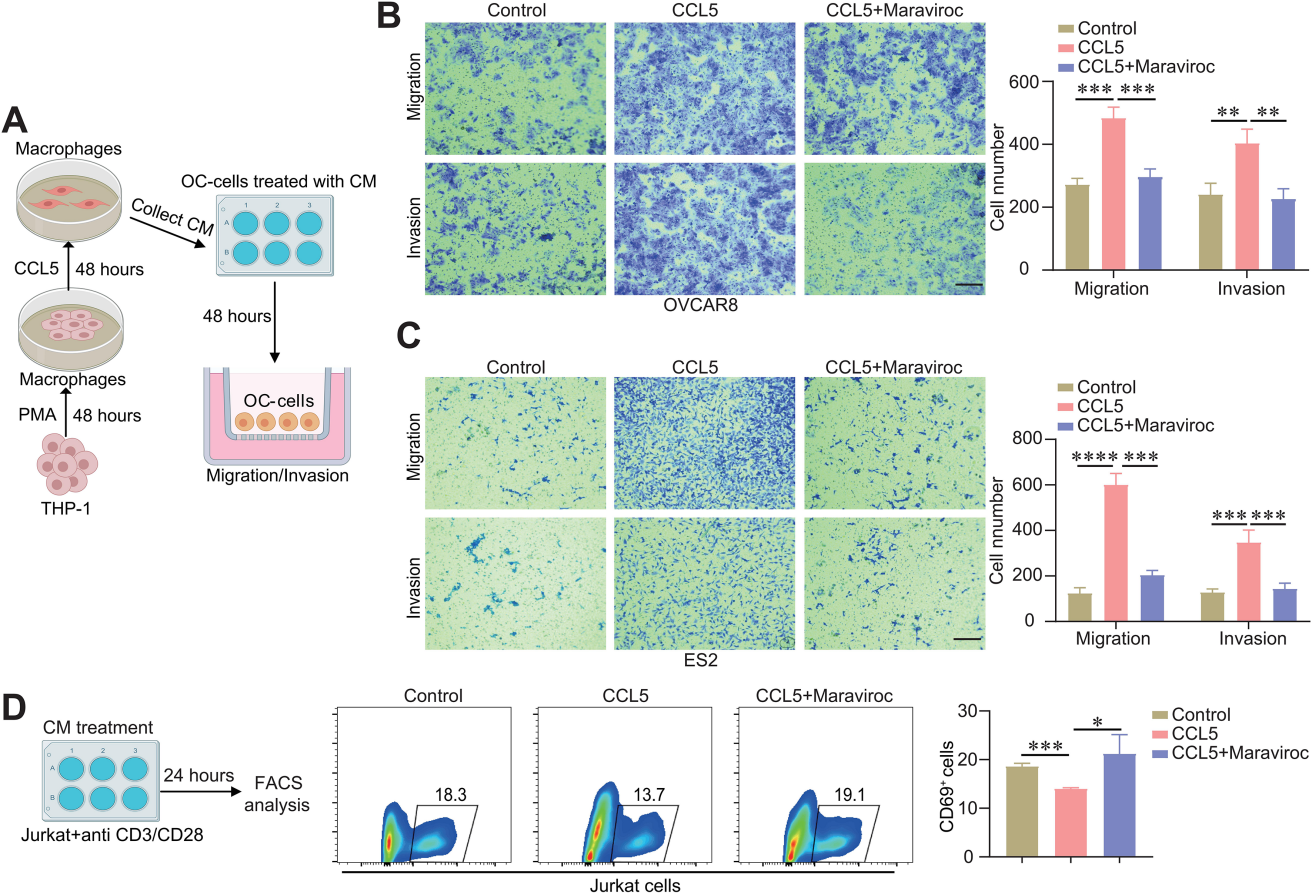
CCL5-mediated M2c macrophages promote tumor progression and immune suppression. **(A)** Schematic diagram of the Transwell assay using macrophage-CM to treat tumor cells. **(B–C)** Migration and invasion assays of OVCAR8 **(B)** and ES2 **(C)** cells treated with CM under the indicated conditions. Representative images of migrated cells (top) and invaded cells (bottom) are shown. Scale bar, 100 μm. **(D)** Flow cytometric assessment of CD69 expression in activated Jurkat cells following treatment with CM derived from macrophages under the indicated conditions (left), with quantitative analysis of CD69 expression levels in each group (right). Data are presented as mean ± SD. Statistical analysis was performed using one-way ANOVA followed by Tukey’s multiple comparisons test **(B, C)** or unpaired two-tailed Student’s t-test **(D)**. *p < 0.05, **p < 0.01, ***p < 0.001, ****p < 0.0001.

To further investigate the functional consequences of CCL5-induced M2c macrophage polarization in OC, we next evaluated its impact on tumor progression and immune suppression. Macrophages were pretreated with PMA and CCL5 or maraviroc for 48 hours, after which CM was collected and applied to OC cells to evaluate migration and invasion (**Figure 6A**). The Transwell experiment showed that the CM of CCL5-treated macrophages significantly enhanced the migration and invasion of OC cells, while treatment with the FDA-approved CCR5 antagonist maraviroc significantly weakened these effects of CCL5-treated macrophages on tumor cells (**Figures 6B, C**).

To further assess the immunomodulatory effects of CCL5-induced M2c macrophages, Jurkat T cells were cultured with CM derived from macrophages subjected to the indicated treatments, and T-cell activation was evaluated by measuring the expression of the early activation marker CD69. Flow cytometric analysis showed that CM from CCL5-treated macrophages significantly reduced CD69 expression in Jurkat cells compared with the control group, indicating impaired T-cell activation. In contrast, treatment with the CCR5 antagonist maraviroc partially restored CD69 expression, suggesting that blockade of the CCL5–CCR5 axis alleviates the immunosuppressive effects mediated by CCL5-induced M2c macrophages (**Figure 6D**). Taken together, these results demonstrate that CCL5-induced M2c macrophages contribute to the establishment of a pro-tumorigenic and immunosuppressive microenvironment in OC.

### 3.7 Clinical significance of LILRB4 expression in OC

**Figure 7:**
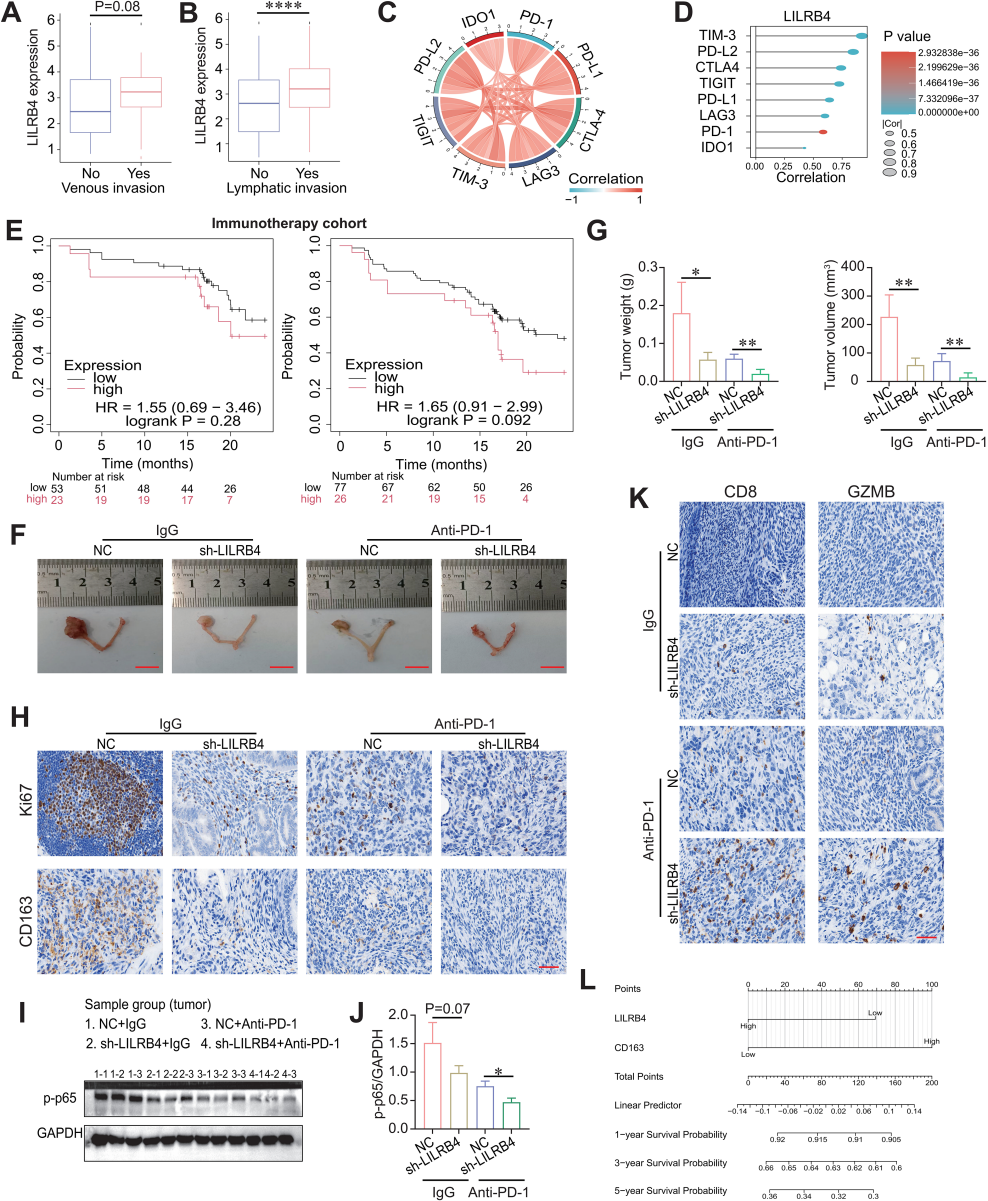
Clinical significance of LILRB4 expression in OC. **(A–B)** Differences in LILRB4 expression levels were compared among subgroups with venous invasion **(A)** and lymphatic invasion **(B)** in the TCGA-OV cohort. **(C–D)** Correlation analysis between LILRB4 and immune checkpoint molecules in the TCGA-OV cohort, presented as a chord diagram **(C)** and a lollipop plot **(D)**. **(E)** Kaplan–Meier survival analysis of patients from the immunotherapy cohort (esophageal adenocarcinoma treated with immune checkpoint inhibitor-based therapy). OS (left) and PFS (right) were compared between tumors with high and low LILRB4 expression. Patients were stratified according to the optimal cutoff determined by the Kaplan–Meier Plotter platform. Statistical significance was assessed using the log-rank test. **(F)** Representative images of orthotopic ID8 tumors in C57BL/6 mice (n = 4 per group). **(G)** Comparison of tumor weight (left) and volume (right) among different groups of C57BL/6 mice (n = 4 per group). **(H)** IHC analysis of Ki-67 and CD163 expression in tumor tissues (n = 3). All images were captured at the same magnification; scale bar = 100 μm. **(I–J)** WB analysis of p-p65 expression in tumor tissues (I, n = 3) and corresponding quantitative analysis **(J)**. **(K)** IHC analysis of CD8 and GZMB expression in tumor tissues (n = 3). All images were captured at the same magnification; scale bar = 100 μm. **(L)** A nomogram was constructed based on independent prognostic factors for overall survival. Data are presented as mean ± SD. Statistical analysis was performed using an unpaired two-tailed Student’s t-test. *p < 0.05, **p < 0.01, ****p < 0.0001.

To comprehensively assess the clinical significance of LILRB4 expression in OC, we analyzed its correlation with clinicopathological features, including age, race, histologic grade, venous invasion, and lymphatic invasion. We found that high LILRB4 expression was positively associated with venous invasion and lymphatic invasion, but showed no significant association with other clinicopathological features (**Figures 7A, B**; **Supplementary Table 3**). Given the role of LILRB4 in macrophage-mediated tumor progression, we further explored its impact on tumor immune evasion in the TIME and its association with the efficacy of immune checkpoint therapy in OC. To characterize its immunological relevance, we first analyzed the correlation between LILRB4 expression and immune checkpoint molecules. As shown in **Figures 7C, D**, high LILRB4 expression was strongly positively correlated with multiple immune checkpoint molecules, including PD-1, CTLA-4, and LAG3 (R > 0.3, p < 0.0001). To further explore its clinical implications, we observed that patients with high LILRB4 expression had significantly poorer survival after receiving immunotherapy compared with those with low LILRB4 expression (**Figure 7E**).

**Figure 8:**
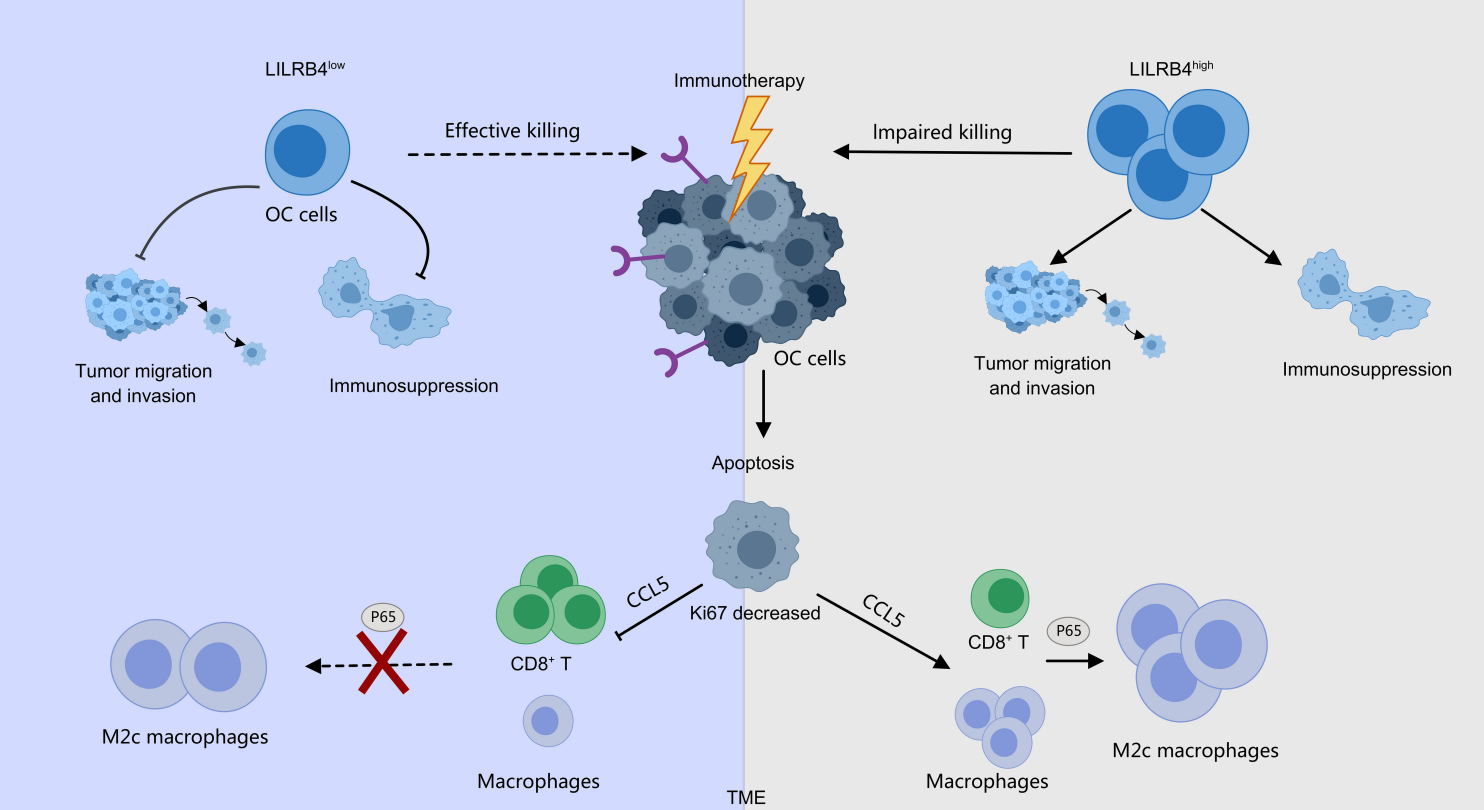
Graphic abstract of LILRB4 in modulating the TIME in OC. An increase in LILRB4 expression promotes tumor development and acts as a key regulator of immune remodeling. It enhances CCL5 secretion, thereby activating NF-κB signaling in macrophages and promoting M2c macrophage polarization. This immunosuppressive reprogramming facilitates tumor growth and progression within the TME. Therefore, targeting LILRB4 may represent a promising therapeutic strategy to improve immunotherapy efficacy, enhance antitumor immunity, and achieve better clinical outcomes in OC.

Then, we experimentally validated the *in vivo* functional role of LILRB4 and assessed whether LILRB4 knockdown could enhance sensitivity to immunotherapy. We established an orthotopic ID8 tumor xenograft mouse model and treated tumor-bearing mice with anti–PD-1 therapy. We found that either LILRB4 knockdown or PD-1 blockade alone inhibited tumor growth; however, the combination treatment produced the most pronounced anti-tumor effect (**Figures 7F, G**). This enhanced therapeutic efficacy was accompanied by decreased tumor cell proliferation and reduced M2 macrophage infiltration, as evidenced by lower Ki-67 and CD163 expression (**Figure 7H**). Moreover, WB analysis of tumor tissues confirmed the *in vitro* findings, showing that LILRB4 knockdown suppressed M2 macrophage polarization, accompanied by decreased p-p65 expression (**Figures 7I, J**). Consistent with the proposed LILRB4–CCL5 signaling axis, qRT-PCR analysis further demonstrated that Ccl5 expression was significantly decreased in tumors with LILRB4 knockdown (**Supplementary Figure 5A**). To further characterize the immune status of the TME, we assessed T-cell activation by measuring the expression of Ifng and Tnf, as well as T-cell exhaustion by evaluating Pdcd1 (PD-1), Lag3, and Tim-3. LILRB4 knockdown, particularly when combined with anti–PD-1 treatment, resulted in increased expression of Ifng and Tnf and decreased expression of Pdcd1, Lag3, and Tim-3, indicating enhanced T-cell effector function and reduced T-cell exhaustion (**Supplementary Figures 5B, C**). Consistently, increased CD8^+^ T-cell infiltration and elevated GZMB expression further supported enhanced cytotoxic T-cell activity within the TME (**Figure 7K**). Based on these findings, we developed a prognostic nomogram integrating these biomarkers to quantitatively predict survival outcomes in OC (**Figure 7L**). Collectively, these findings indicate that LILRB4 promotes an immunosuppressive TME and contributes to immunotherapy resistance in OC, highlighting LILRB4 as a potential therapeutic target. Additionally, **Figure 8** presents a comprehensive schematic overview summarizing the key pathways, biological processes, and mechanistic insights uncovered in this study.

## 4 Discussion

In this study, we systematically investigated the expression pattern, biological function, and clinical significance of LILRB4 in OC, and uncovered its critical role in shaping an immunosuppressive TME. Our findings demonstrate that LILRB4 is significantly upregulated in OC and is closely associated with poor prognosis. Functionally, LILRB4 promotes tumor progression by enhancing malignant phenotypes of OC cells and by remodeling the immune microenvironment, particularly through regulating macrophage infiltration and polarization. Mechanistically, we further identified a LILRB4–CCL5–NF-κb axis that drives M2c macrophage polarization and tumor progression, and contributes to immunotherapy resistance.

We first showed that LILRB4 is aberrantly overexpressed in OC across multiple datasets, including TCGA, GEO, and our clinical samples, at both mRNA and protein levels. Importantly, high LILRB4 expression was consistently associated with worse OS and PFS, highlighting its potential as a prognostic biomarker. These findings are in line with previous studies reporting the immunosuppressive role of LILRB4 in other malignancies, suggesting that LILRB4 may represent a conserved regulator of tumor progression across cancer types (46–48). Functionally, our *in vitro* and *in vivo* experiments demonstrated that LILRB4 directly contributes to the malignant behavior of OC cells. Knockdown of LILRB4 significantly inhibited proliferation, migration, and invasion, and suppressed tumor growth in orthotopic mouse models. These results indicate that LILRB4 is not merely a biomarker but also an active driver of tumor progression. Notably, the reduction in Ki-67 expression further supports its role in promoting tumor cell proliferation.

Beyond its tumor-intrinsic effects, a key finding of this study is the role of LILRB4 in modulating the TIME. Bioinformatics analyses revealed that LILRB4 expression is strongly associated with immune-related pathways and increased immune cell infiltration, particularly macrophages. Single-cell RNA sequencing data further confirmed that LILRB4 is predominantly enriched in monocyte/macrophage populations. Both human tissues and mouse models consistently showed that high LILRB4 expression is associated with increased macrophage infiltration, suggesting that LILRB4 plays a crucial role in recruiting or maintaining macrophages within the TME. Importantly, we demonstrated that LILRB4 not only promotes macrophage infiltration but also drives their polarization toward the tumor-promoting M2c phenotype. Elevated LILRB4 expression was associated with increased M2 macrophage enrichment and higher expression of M2c markers, while its knockdown suppressed M2c polarization both at the transcriptional and protein levels. Given that M2c macrophages are known to support tumor growth, angiogenesis, and immune evasion, these findings provide a mechanistic explanation for the association between LILRB4 and poor prognosis in OC (49, 50).

Mechanistically, we have discovered that CCL5 is a key regulatory factor for LILRB4-induced M2c polarization of macrophages. Relevant analyses and functional experiments consistently indicate that LILRB4 regulates the release of CCL5 by tumor cells, thereby activating the NF-κB signaling pathway. This is confirmed by the increase in p-p65 levels, and pharmacological inhibition of NF-κB reversed the M2c polarization. These results suggest that LILRB4 functions upstream of CCL5 to regulate the function of macrophages, highlighting this crucial signaling axis in the TME. Additionally, M2c macrophages treated with CCL5 also promote tumor progression and immunosuppression. Functional experiments demonstrated that CCL5 not only enhanced M2c polarization but also promoted the migration and invasion of OC cells through this process and inhibited the early activation of T cells. Importantly, using marvastir to block the CCL5 receptor CCR5 significantly weakened these effects, indicating that the CCL5-CCR5 axis is a key downstream effector factor of the LILRB4 signaling pathway. This discovery provides a potential therapeutic strategy for disrupting the pro-tumor interaction between tumor cells and macrophages.

Clinically, we further demonstrated that high LILRB4 expression is associated with more aggressive disease features and is positively correlated with multiple immune checkpoint molecules such as PD-1, CTLA-4, and LAG3. Notably, patients with high LILRB4 expression exhibited poorer responses to immunotherapy, suggesting that LILRB4 contributes to immune evasion. Importantly, *in vivo* experiments showed that LILRB4 knockdown significantly enhanced the efficacy of anti–PD-1 therapy. This was accompanied by increased expression of T-cell activation-associated genes (Ifng and Tnf) and decreased expression of T-cell exhaustion markers (Pdcd1, Lag3, and Tim-3), indicating restored T-cell effector function and reduced exhaustion. Consistently, LILRB4 knockdown also increased CD8^+^ T cell infiltration and elevated GZMB expression, reflecting enhanced cytotoxic T cell activity within the TME. These findings highlight the potential of targeting LILRB4 to overcome immunotherapy resistance and improve treatment outcomes.

Despite these strengths, several limitations should be acknowledged. First, although our study integrates multi-omics data and experimental validation, larger prospective cohorts are needed to further validate the clinical utility of LILRB4 as a prognostic and predictive biomarker. Particularly, due to the limited availability of OC cases and the lack of an independent cohort with complete clinicopathological and follow-up information at our institution, external validation of the prognostic model could not be performed in the present study. Accordingly, the nomogram requires further validation in larger, independent cohorts prior to clinical application. Second, while we identified the CCL5–NF-κB axis as a key mechanism, other signaling pathways may also be involved in LILRB4-mediated immune regulation and warrant further investigation. Third, the detailed molecular interactions between tumor cells and macrophages remain to be fully elucidated.

## 5 Conclusion

In conclusion, our study demonstrates that LILRB4 acts as a critical regulator of tumor progression and immune remodeling in OC by promoting M2 macrophage polarization through the CCL5–NF-κB axis. Targeting LILRB4 may represent a potential therapeutic strategy to improve immunotherapy efficacy and clinical outcomes in OC.

## Data Availability

The raw data supporting the conclusions of this article will be made available by the authors, without undue reservation.
