# Supplementary material for: Identification of LILRB4 as a regulator of M2c macrophages and a potential immunotherapeutic target in ovarian cancer
Source: Front Immunol. 2026 Jul 27;17:1866073. doi: 10.3389/fimmu.2026.1866073 (PMC13454356; doi:10.3389/fimmu.2026.1866073)
Supplement: Supplementary file 1 [file DataSheet1.docx]

**Supplementary Materials for**

**Identification of LILRB4 as a Regulator of M2c Macrophages and a Potential Immunotherapeutic Target in Ovarian Cancer**

**This file includes:**

**Supplementary Table S1 to S3**

**Supplementary Figure S1-S5 and Figure Legends**

**Supplementary Table S1 qRT-PCR primer list**

| Gene name | Species | Forward Sequence | Reverse Sequence |
| --- | --- | --- | --- |
| GAPDH | Human | AAAGGCATTCTTCACCTGCTCC | GCCATCACGCCACAGTTTC |
| LILRB4 | Human | CATCCATGACAGAGGACTATGC | GGGCTGAAAGGGTGGGTTTA |
| F4/80 | Mouse | CTCAGTCTGCACCAATATCCTG | CCACAGAGTTAGAGCAGTTGGAA |
| β-actin | Mouse | GTGACGTTGACATCCGTAAAGA | GCCGGACTCATCGTACTCC |
| IL-10 | Human | CTGAGAACCAAGACCCAGACA | AAAGGCATTCTTCACCTGCTCC |
| TGFB1 | Human | CACTCTCAAACCTTTACGAGACC | CGTTGCTAGGGGCGAAGATG |
| CD163 | Human | TTTGTCAACTTGAGTCCCTTCAC | TCCCGCTACACTTGTTTTCAC |
| CCL2 | Human | AGCAGCAAGTGTCCCAAAGA | TTGGGTTTGCTTGTCCAGGT |
| CCL5 | Human | CCAGCAGTCGTCTTTGTCAC | CTCTGGGTTGGCACACACTT |
| CCL18 | Human | GTTGACTATTCTGAAACCAGCCC | GTCGCTGATGTATTTCTGGACCC |
| CCL22 | Human | ATCGCCTACAGACTGCACTC | GACGGTAACGGACGTAATCAC |
| CXCL12 | Human | CCGCGCTCTGCCTCAGCGACGGGAAG | CTTGTTTAAAGCTTTCTCCAGGTACT |
| CXCL8 | Human | TTTTGCCAAGGAGTGCTAAAGA | AACCCTCTGCACCCAGTTTTC |
| Ccl5 | Mouse | TTTGCCTACCTCTCCCTCG | CGACTGCAAGATTGGAGCACT |
| Ifng | Mouse | GCCACGGCACAGTCATTGA | TGCTGATGGCCTGATTGTCTT |
| Tnf | Mouse | CAGGCGGTGCCTATGTCTC | CGATCACCCCGAAGTTCAGTAG |
| Pdcd1 | Mouse | CAGCTTGTCCAACTGGTCG | GCTCAAACCATTACAGAAGGCG |
| Lag3 | Mouse | CTGGGACTGCTTTGGGAAG | GGTTGATGTTGCCAGATAACCC |
| Tim-3 | Mouse | TCAGGTCTTACCCTCAACTGTG | GGCATTCTTACCAACCTCAAACA |

**Supplementary Table S2 Flow cytometry antibodies and reagents**

| Antibodies/ reagents | Source | Identifier |
| --- | --- | --- |
| FITC anti-human CD11b | Biolegend | Cat#982614 |
| APC Anti-Human CD163 Antibody | Biolegend | 333609 |
| FITC Anti-Human CD69 Antibody | Elabscience Biotechnology | E-AB-F1138C |
| Immobilon®-P PVDF Membrane | Sigma-Aldrich | IPVH00010 |
| Nonfat milk | Epizyme | PS112 |
| Tris - Buffered Saline with Tween (TBST) | Epizyme | PS103 |
| InVivoMAb anti-mouse PD-1 (CD279) | STARTER | Cat#S0B0594 |
| Phorbol 12-myristate 13-acetate | MedChemExpress | Cat#HY-18739 |
| CCL5 protein, Human | MedChemExpress | Cat#HY-P70450 |
| Maraviroc | MedChemExpress | Cat#HY-13004 |

**Supplementary Table S3 Based on the TCGA-OV cohort, the association between LILRB4 expression levels and patients’ clinicopathological parameters (race, age, and** **histologic grade) was investigated**

| Characteristics | Low expression of LILRB4 | High expression of LILRB4 | P value |
| --- | --- | --- | --- |
| n | 190 | 191 |  |
| Race, n (%) |  |  | 0.150 |
| Asian & Black or African American | 23 (6.3%) | 14 (3.8%) |  |
| White | 164 (44.7%) | 166 (45.2%) |  |
| Age, n (%) |  |  | 0.282 |
| <= 60 | 99 (26%) | 110 (28.9%) |  |
| > 60 | 91 (23.9%) | 81 (21.3%) |  |
| Histologic grade, n (%) |  |  | 0.467 |
| G1&G2 | 25 (6.7%) | 21 (5.7%) |  |
| G3&G4 | 158 (42.6%) | 167 (45%) |  |

**Supplementary Figure S1**

**Figure S1 DNA CNV of LILRB family genes in OC based on the TCGA dataset.**

**Supplementary Figure S2**

**Figure S2 Enrichment analysis of expression profile characteristics of LILRB4 in OC.**

(A-C) Analyses were performed using The LinkedOmics (http://www.linkedomics.org/) database. (A) The volcano plot illustrates the correlation of co-expressed genes with LILRB4 in OC. (B-C) The heatmap displays the top 50 genes in EOC that exhibit the strongest positive (left) and negative (right) correlations with LILRB4. (D) Gene Ontology enrichment analyses of LILRB4 co-expressed genes.

**Supplementary Figure S3**

**Figure S3 LILRB4 is associated with macrophages in the OC immune microenvironment.**

(A) Analysis using the TIMER database showed that LILRB4 expression was significantly positively correlated with macrophage infiltration levels (left) and the tumor-associated macrophage marker CD68 (right). (B) LILRB4 expression was significantly positively correlated with M2 macrophage markers TGFβ1 (left) and IL-10 (right).

**Figure S4 LILRB4 levels are positively correlated with CCL5 in OC.**

The levels of CCL5 in the control group and LILRB4-knockdown OC cell lines (OVCAR8, left figure; ES2, right figure) were detected by ELISA. Data are presented as mean ± SD (n = 3). Statistical significance was determined using an unpaired two-tailed Student’s t-test. *p < 0.05.

**Figure S5 LILRB4 knockdown suppresses CCL5 expression and reverses T-cell dysfunction in vivo.**

(A) qRT-PCR analysis of Ccl5 expression in tumor tissues from different treatment groups. (B) qRT-PCR analysis of T-cell activation-associated genes (Ifng and Tnf) in tumor tissues from different treatment groups. (C) qRT-PCR analysis of T-cell exhaustion-associated genes (Pdcd1, Lag3, and Tim-3) in tumor tissues from different treatment groups. Data are presented as mean ± SD. Statistical analysis was performed using an unpaired two-tailed Student’s t-test. *p < 0.05, **p < 0.01, ***p < 0.001, ****p < 0.0001.
